# Association between Neuroticism and Emotional Face Processing

**DOI:** 10.1038/s41598-017-17706-2

**Published:** 2017-12-15

**Authors:** Silke Klamer, Lena Schwarz, Oliver Krüger, Katharina Koch, Michael Erb, Klaus Scheffler, Thomas Ethofer

**Affiliations:** 1 Department of Neurology and Epileptology, Hertie Institute for Clinical Brain Research, University of Tübingen, Tübingen, Germany; 2 Department for Biomedical Magnetic Resonance, University of Tübingen, Tübingen, Germany; 3 Department of Psychiatry and Psychotherapy, University of Tübingen, Tübingen, Germany; 4 Department of Neuroradiology, University of Tübingen, Tübingen, Germany; 5 Max-Planck-Institute for Biological Cybernetics, Tübingen, Germany

## Abstract

Neuroticism is one of the “Big Five” personality factors and is characterized by a tendency to experience negative affect. We aimed to investigate how neuroticism influences the neural correlates for processing of emotional facial expressions. 68 healthy participants were presented with emotional dynamic facial stimuli, i.e. happy, neutral or angry, during functional MRI. Brain activations for the contrasts emotional vs. neutral, happy vs. neutral and angry vs. neutral were correlated with individuals’ neuroticism scores as obtained by the NEO Five Factor Inventory questionnaire and additionally investigated for gender differences. The bilateral medial temporal gyrus (MTG) was identified as key region in the processing of emotional faces and activations within this region correlated with individual neuroticism scores. Although female participants showed significantly stronger activation differences between emotional and neutral facial expressions in the left MTG, the correlation between activation and neuroticism scores did not show any significant gender differences. Our results offer for the first time a biological correlate within the face processing network for enhanced reactivity of neurotic individuals to emotional facial expressions which occurs similarly for both male and female participants.

## Introduction

According to the so-called “Big Five” personality model, the human personality can be described by five different personality traits: Neuroticism, Extraversion, Openness, Agreeableness, and Conscientiousness^1^. Of special interest in clinical neuroscience is the factor neuroticism due to its association with certain psychiatric diseases such as anxiety disorders, depression, and substance use disorders^2,3^. Neuroticism is characterized by the tendency to experience negative affect or dissatisfaction, to worry, and to experience anxiousness^4–6^. Several neuroimaging studies have been performed to investigate the neural correlates of neuroticism using different stimulus categories. Correlations between neuroticism scores and brain activation have been shown for the amygdala, the middle temporal gyrus (MTG), the middle frontal gyrus (MFG) and the dorsomedial prefrontal cortex (DMPFC) using emotional pictures as stimuli^6–10^ and for the amygdala, the MTG, temporal pole, left MFG, DMPFC, bilateral parietal cortex, visual cortex, and the fusiform gyrus using static emotional faces as stimuli^11–17^. Using a prisoner dilemma task, Feng, *et al*.^18^ were able to correlate neuroticism with the neural response to negative and positive social interaction in the anterior cingulate cortex/medial prefrontal cortex and the insula, respectively.

Correct interpretation of facial expressions is a prerequisite for effective social interaction. Therefore, face perception has been the subject of numerous neuroimaging studies in the past years, leading to the discovery of a distributed face network of which the occipital face area (OFA), the fusiform face area (FFA), and the posterior superior temporal sulcus (pSTS) are the core areas^19–22^. While the OFA and the FFA are hypothesized to be involved mainly in the processing of invariant facial aspects, such as identity or gender^20,23^, the cortex adjacent to the pSTS is thought to process changeable aspects like emotional facial expressions^20,24,25^.

Bridging the gap between basic functions underlying social interaction and individual personality traits, we aimed to identify the neural correlates of neuroticism with regards to the processing of emotional facial expressions using functional MRI in a large cohort of healthy participants. Subjects were presented with video clips of emotional face stimuli, i.e. happy or angry expressions, as such stimuli have a higher ecological validity than static images of faces. Furthermore, it has been shown that face-selective regions can be more robustly identified using dynamic compared to static stimuli^26–28^. To identify brain areas associated with certain personality traits, especially neuroticism, activations were correlated with participants’ personality scores as obtained by the NEO Five Factor Inventory questionnaire^29^. We hypothesized that activity of areas supporting processing of dynamic facial information, i.e. the cortical areas adjacent to the pSTS, is correlated with neuroticism. Finally, electrophysiological studies using electroencephalography^30^ or magnetencephalography^31^ as well as neuroimaging studies point to gender differences in face processing^32,33^. Therefore, we also examined whether relationships between neuroticism and activation to emotional faces are different or similar in male and female participants.

## Results

### Behavioral data

Intercorrelations between the five personality factors are presented in Table 1. The only intercorrelation which was significant after Bonferroni correction was a negative correlation between neuroticism and extraversion (r = 0.59, p < 0.001). Concerning gender differences, women scored significantly higher on neuroticism (20.7 ± 1.41 versus 16.7 ± 1.41, T(66) = −2.03, two-tailed p < 0.05) and conscientiousness (35.4 ± 0.96 versus 31.8 ± 1.32, T(66) = −2.23, two-tailed p < 0.05) than men. The task of the participants was to identify the gender of the presented face stimuli. They responded in 99.9% of the trials (mean reaction time = 1.98 ± 0.02 s) and correctly identified the gender of the presented actors in 96.0 ± 0.7% of the trials indicating good attention to the presented stimulus material throughout fMRI scanning. There were no significant differences in hit rates or reaction time between female and male participants (both T(66) < 0.70, both two-tailed p > 0.39).

**Table 1:** Intercorrelations (Pearson’s r) between the five personality factors across the 68 participants.

	Extraversion	Openness	Agreeableness	Conscientiousness
Neuroticism	−0.59*	−0.08	−0.24	−0.26
Extraversion		0.16	0.21	0.33
Openness			0.21	0.07
Agreeableness				0.24

*p < 0.05, Bonferroni corrected.

### fMRI activation

**Figure 1:**
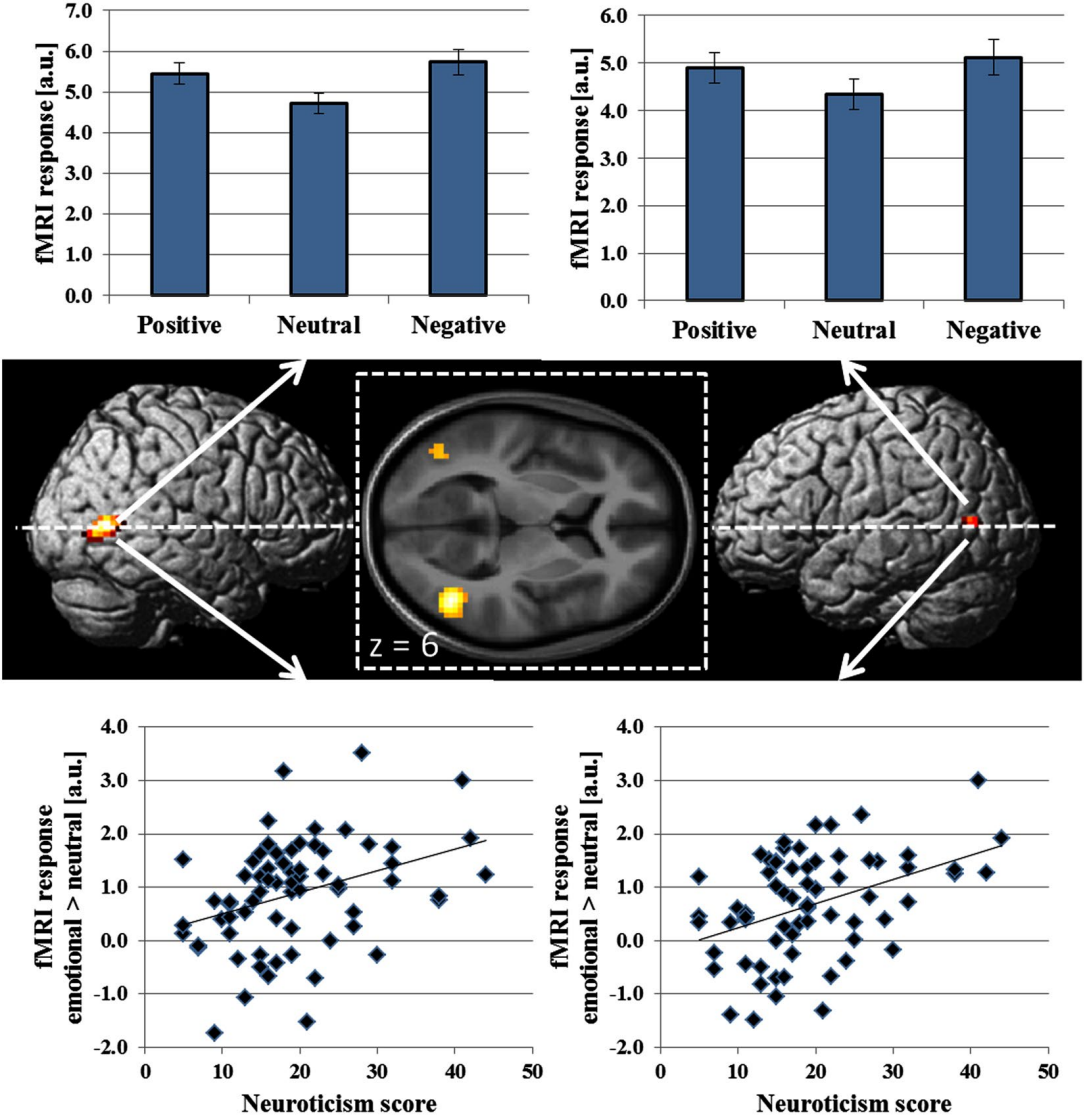
Brain activation to video clips displaying significantly stronger activation (p < 0.05, FWE corrected at voxel level) to emotional versus neutral facial expressions are rendered on the surface of the standard SPM brain template as well as on a transversal slice (z = 6) of the mean normalized brain of the study participants. Activations (beta estimates) ± standard error to happy (positive), neutral, and angry (negative) facial expressions are shown in the upper panels. Correlation analyses between activation differences to emotional versus neutral facial expressions in right and left MTG and neuroticism are shown in the lower panels.

The first step of the fMRI data analysis aimed to identify brain areas involved in processing facial emotions. To this end, we compared activation to video clips displaying emotional versus neutral facial expressions which yielded significant differences (voxel-wise corrected height threshold: p < 0.05, see methods) in bilateral MTG (see Table 2 and Fig. 1). This effect was due to stronger activation to both happy and angry as compared to neutral facial expressions (see Fig. 1, upper panels). A direct gender comparison revealed that in the left MTG, female participants exhibited a larger difference for emotional versus neutral stimuli than male participants (T(66) = −2.74, two-tailed p < 0.01). In the right MTG, no significant difference between male and female participants was found (T(66) = −1.00, two-tailed p = 0.32).

**Table 2:** Brain areas showing significantly stronger activation to video clips showing emotional versus neutral facial expressions.

Brain area	MNI coordinates	Z score	cluster size
Right middle temporal gyrus	x = 45; y = −63; z = 3	7.12*	104
Left middle temporal gyrus	x = −45; y = −69; z = 6	5.03*	17

*p < 0.05, FWE corrected at voxel level across the whole brain.

In the second step, we tested whether activation in the areas defined by the contrast emotional versus neutral facial expressions correlated with neuroticism scores. A significant correlation was found for both the cluster in right (r = 0.36, 95% confidence interval: 0.13 < r < 0.55, p = 0.003) and left (r = 0.42, 95% confidence interval: 0.20 < r < 0.60; p < 0.001) MTG (see Fig. 1, lower panels) which survived Bonferroni correction for ten statistical tests (two regions x five personality factors). No significant correlation was found for any of the other four personality factors (see Table 3).

**Table 3:** Correlation between activation to emotional versus neutral facial expressions and personality factors within right and left middle temporal gyrus (MTG).

Brain area	Neuroticism	Extraversion	Openness	Agreeableness	Conscientiousness
Right MTG	r = 0.36*	r = −0.18	r = 0.14	r = 0.06	r = 0.02
Left MTG	r = 0.42*	r = −0.22	r = 0.10	r = 0.03	r = 0.09

*p < 0.05, Bonferroni corrected, MTG = middle temporal gyrus.

In the third step, we investigated potential effects of the participants’ gender and valence of the stimuli (i.e. positive versus negative emotional information). Separate analysis revealed significant correlations in bilateral MTG both for female (right MTG: r = 0.30, 95% confidence interval: 0.02 < r < 0.55, p = 0.040; left MTG: r = 0.37, 95% confidence interval: 0.08< r < 0.59, p = 0.12) and male (right MTG: r = 0.47, 95% confidence interval: 0.06 < r < 0.74, p = 0.028; left MTG: r = 0.43, 95% confidence interval: 0.01 < r < 0.72, p = 0.046) participants with no significant differences between groups. Valence specific correlation analysis revealed significant correlations between neuroticism and activation of the right MTG to positive versus neutral stimuli (r = 0.26, 95% confidence interval: 0.02 < r < 0.47, p = 0.031) and negative versus neutral stimuli (r = 0.36, 95% confidence interval: 0.11 < r < 0.54, p = 0.003) without significant differences between conditions (p = 0.18). In the left MTG, a significant correlation was found for activation of negative versus neutral stimuli (r = 0.47, 95% confidence interval: 0.22 < r < 0.61, p < 0.001), which marginally failed to reach significance for positive versus neutral stimuli (r = 0.22, 95% confidence interval: −0.02 < r < 0.43, p = 0.07). The difference between the two conditions was significant (p = 0.02) for the left MTG.

In the fourth step, we examined whether removal of variance correlating with the other four personality factors by means of simple regression impacts the observed results obtained for neuroticism. Removal of variance correlating with extraversion reduced the correlation coefficients for the right (r = 0.25, 95% confidence interval: 0.01 < r < 0.46, p = 0.037) and left MTG (r = 0.29, 95% confidence interval: 0.05 < r < 0.49, p = 0.016). Openness, agreeableness, and conscientiousness had no considerable effect on correlation between activation to emotional versus neutral facial expressions and neuroticism which ranged between r = 0.36 and r = 0.37 in the right MTG and r = 0.42 and r = 0.44 in the left MTG (all p < 0.003). Similarly, the correlation coefficients remained significant after removing the conjoint effect of the other four personality factors by means of multiple regression analysis (right MTG: r = 0.38, p = 0.002, left MTG: r = 0.49, p < 0.001).

**Figure 2:**
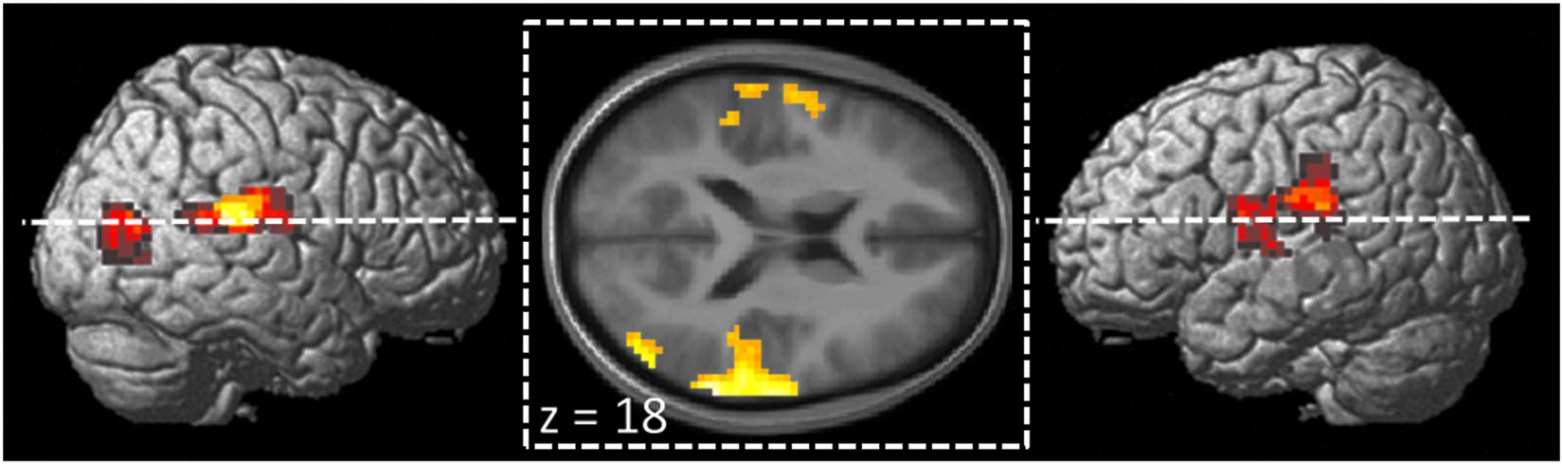
Correlation between activation to emotional versus neutral facial expressions and neuroticism scores revealed significant correlations in bilateral TPJ including superior temporal gyrus and supramarginal gyrus as well as right MTG (p < 0.05, FWE corrected at cluster level (k > 90 voxels)). Results are rendered on the surface of the standard SPM brain template as well as on a transversal slice (z = 18) of the mean normalized brain of the study participants.

Finally, we conducted whole-brain correlation analyses between brain activation to emotional versus neutral facial expressions and the five personality factors. This exploratory approach was carried out on a less conservative threshold (voxel-wise uncorrected height threshold p < 0.001, correction for multiple comparison at cluster level, see methods) to make these results accessible to future meta-analyses on associations between personality factors and brain activity. This revealed a significant correlation between neuroticism scores and activation to emotional versus neutral facial expressions in bilateral temporoparietal junction (TPJ) including superior temporal gyrus and supramarginal gyrus as well as right MTG (see Table 4 and Fig. 2). In left MTG, this analysis failed to reach significance at cluster level (peak MNI coordinates: x = −42, y = −63, z = 6; Z = 3.99; k = 41 voxels). For agreeableness, a significant correlation with activation to emotional versus neutral facial expressions was found in left medial prefrontal cortex (MNI coordinates: x = −15, y = 46, z = 6; Z = 3.79; k = 96 voxels, p < 0.05, corrected). No significant correlations were found for any of the other three personality factors.

**Table 4:** Whole-brain analysis between activation to emotional versus neutral facial expressions and neuroticism scores.

Brain area	MNI coordinates	Z score	cluster size
Right superior temporal gyrus/supramarginal gyrus	x = 66 y = −42 z = 15	4.88*	303
Right middle temporal gyrus/middle occiptal gyrus	x = 48 y = −78 z = 9	4.46*	96
Left superior temporal gyrus/supramarginal gyrus	x = −66 y = −63 z = 6	4.13*	235

*p < 0.05, corrected at cluster level (k > 90 voxels).

## Discussion

In our study, we investigated the impact of neuroticism on processing of dynamic emotional facial stimuli. In agreement with the face model of Haxby and colleagues^20^, bilateral MTG areas adjacent to the pSTS responded stronger to emotional than to neutral dynamic facial expressions. This enhanced responsiveness to facially displayed emotions correlated significantly with neuroticism scores suggesting this area as key structure underlying increased perception of affect in neurotic individuals.

Here, we decided to define brain areas processing facial emotions by directly contrasting activations to dynamic facial emotional and neutral expressions. This approach yields better emotion specific results than application of an fMRI localizer which compares activation to different classes of stimuli (e.g. faces versus objects) as such comparisons are burdened with differences in a variety of low-level features (e.g. size, shape, contrast) and associated higher-level processes (e.g. faces, but not object reflect individuals, objects, but not faces are associated with typical movements to use them).

Contrasting emotional with neutral dynamic facial expressions resulted in significantly stronger responses in bilateral MTG adjacent to the pSTS. This area is thought to represent the key structure for processing dynamic facial information^20,24,25,34^ as well as their integration with vocal information^35,36^. Furthermore, this region has been previously described to react preferentially to emotionally salient stimuli^15,37^. In our study, this activation difference was more pronounced and yielded a larger cluster in the right than in the left hemisphere which corroborates previous neuroimaging results^38^. Concerning gender differences, however, the left MTG showed a significantly stronger enhancement of activity in the contrast emotional versus neutral faces in female than in male participants. This probably reflects the fact that the female participants scored higher in neuroticism which is in accordance with the literature^39–42^.

Our results showed that activation in bilateral MTG during the processing of emotional faces correlated positively with neuroticism scores, i.e. the higher the participant’s neuroticism score the stronger the activation in bilateral MTG for both male and female participants. This suggests that the personality factor neuroticism and the processing of salient facial stimuli within the MTG are strongly related and that this mechanism occurs irrespective of gender. For negative stimuli, a positive correlation was found for bilateral MTG. It is well known that neuroticism is particularly associated with an enhanced experience of negative affect^4–6^. Therefore, it is not surprising that the relation between processing of salient facial information and neuroticism is more consistently found for negative information. Hyperreactivity to negative stimuli in affective brain regions, including the MTG, in high neurotic individuals has been described previously and has been attributed to increased arousal by emotional conflict and negative affect^4,6,43–45^ or more intensive visual processing of threat-relevant (face) stimuli^14^. In right MTG, activation to positive stimuli was also correlated with neuroticism indicating that enhanced responsiveness of neurotic individuals to emotional stimuli is not restricted to negative valence. Future studies should investigate whether the effects observed for the emotions happiness and anger occur similarly for other emotions (e.g. sadness, fear, disgust). Furthermore, it should be clarified whether the correlation between neuroticism and responses to emotional stimuli depend on the intensity of the portrayed emotions and whether the effects which we found here for the MTG and faces can be generalized across sensory modalities (i.e., voices or bodies). Corresponding structures for a correlation between neuroticism and responses to emotional voices and bodies would be the temporal voice area^46^ and extrastriate body area^47^ which also show a particular sensitivity for affective information^48,49^. No correlation between amygdala activation and neuroticism scores was found (even at an uncorrected threshold of p < 0.05). The implicit nature of the paradigm employed here might offer an explanation for this finding as a recent resting-state fMRI study^50^ showed a correlation between neuroticism and connectivity of the amygdala with frontal structures involved in explicit evaluation of emotional faces^51^, but not with face processing areas in sensory cortices.

In theory, the “Big Five” factors are conceptually independent dimensions. However, we observed a significant negative correlation between neuroticism and extraversion scores in our cohort. Such intercorrelations make it difficult to fully disambiguate the contribution of single personality factors to brain responses and thus represent a limitation of the current study. However, it should be noted that this negative correlation between neuroticism and extraversion does not reflect a phenomenon which is due to the specific group of participants recruited for this study as this intercorrelation has been reported repeatedly^15,29,42,52,53^. To address this issue, we removed the variance correlating with the other four personality factors in separate simple regression analyses as well as a multiple regression analysis which allows for their combined contribution. In all cases, the correlation between responses in bilateral MTG remained significant indicating that neural activity is modulated primarily by neuroticism irrespective of the other four personality factors.

Our results underline the importance of the bilateral MTG for processing emotional facial stimuli. Critically, correlations of MTG activity with neuroticism scores indicate that neuroticism and neural activity in this region are associated providing a potential neural correlate for increased sensitivity of neurotic individuals to emotional information which occurs similarly in women and men. Thus, we conclude that neuroticism modulates neural activity in MTG during processing of facial emotions. It should be noted that correlations never indicate causality and that the reverse explanation is also possible (i.e. that increased activity within emotion processing areas induces neuroticism). However, given the fact that personality traits are conceptually not restricted to judgment of emotional facial expressions, but cover a much wider range of interpersonal relationships, we deem the first explanation more likely than the second.

## Methods

### Participants

68 volunteers (46 females, 22 males, mean age: 26.0 ± 4.9 years, age range: 18–36 years completed the German version of the NEO Five Factor Inventory (NEO FFI)^29^ before functional magnetic resonance imaging (fMRI). The NEO FFI contains 60 items with 12 statements for each of the five personality factors (Neuroticism, Extraversion, Openness, Agreeableness, and Conscientiousness). None of the participants had a history of neurological or psychiatric disorders or was on any psychotropic medication. All participants were right-handed as determined by the Edinburgh Handedness Inventory^54^ and had normal or corrected to normal vision. The study conformed to the code of Ethics of the World Medical Association (Declaration of Helsinki). The study protocol was approved by the ethics committee of the University of Tübingen and the methods were carried out in accordance with the relevant guidelines and regulations. All participants gave written informed consent prior to participating.

### Stimulus material and experimental design

Short video clips (mean duration: 1384 ms ± 264 ms) depicting the faces of professional actors (5 male, 5 female) speaking German sentences consisting of four words (e.g.”Ich fühle mich ruhig” translation: “I feel calm”) in happy, neutral, or angry tone of voice with a corresponding facial expression were used as stimulus material in an event-related design (for a detailed description of the stimuli, see^55^. These stimuli (20 per emotional category) were presented without auditory stimulation (mute video clips) and the participants were engaged in a gender decision task to keep the participants attentive of the stimuli during scanning. We employed an implicit emotion perception paradigm as this more closely resembles natural situations than paradigms that include explicit instructions to judge emotions. Responses were conveyed via button press via a fiber optic system and recorded by the stimulation software (Software Presentation, Neurobehavioral Systems). Responses had no influence on stimulus presentation and no feedback regarding whether the responses were correct or not was given during the experiment. A black fixation cross was presented during an inter trial interval of varying duration ranging from 8.5 to 15 s (jittered in steps of TR/4).

### Image acquisition

All participants were examined in a 3 Tesla PRISMA scanner (Siemens, Erlangen, Germany) using a 20 channel head coil. Functional imaging data comprised 360 volumes acquired with an echo-planar-imaging sequence covering the whole cerebral cortex (30 axial slices acquired in sequential descending order, slice thickness 4 mm + 1 mm gap, TR = 2 s, TE = 30 ms, voxel size = 3 × 3 × 4 mm, flip angle = 90°). For distortion correction, a fieldmap (TR = 400 ms, TEs = 4.92 and 7.38 ms, slice thickness = 3 mm, flip angle = 60°) was acquired prior to each functional run. A high-resolution T1-weighted anatomical 3D magnetization prepared rapid acquisition gradient echo (MPRAGE, FOV = 256 × 256 mm, 1 mm isotropic voxel size, TR = 2.3 s, TE = 4.18 ms, TI = 900 ms, flip angle = 9°) data set was obtained to enable precise normalization of the functional scans.

### Analysis of questionnaires and behavioral data

Behavioral data are reported as mean ± standard error. Questionnaire data obtained from the NEO FFI were examined for intercorrelations (Pearson’s r) between the five personality factors. To allow for multiple comparisons a Bonferroni correction for ten intercorrelations between the personality factors was employed. Behavioral data (hit rates and reaction times) of the fMRI experiment are reported as mean ± standard error. Responses were classified as hits if the gender was correctly classified within 5 seconds after stimulus onset. Correlations between fMRI activations and personality scores are reported including 95% confidence intervals.

### Analysis of fMRI data

The images were analyzed using statistical parametric mapping software (SPM12, Wellcome Department of Imaging Neuroscience, London, UK). The first five functional volumes were discarded to allow for T1 equilibrium. Preprocessing included correction for differences in slice acquisition time with the middle slice as reference, realignment and unwarping to correct for movement as well as static and movement dependent field distortions^56^, coregistration to the high-resolution anatomical image, normalization^57^ to Montreal Neurological Institute (MNI) space (resampled voxel size: 3 × 3 × 3 mm), and spatial smoothing with an isotropic Gaussian filter of 8 mm full width at half maximum. Event-related responses were analyzed using a general linear model with three regressors for videos displaying happy, angry, and neutral emotional expressions using a stick function convolved with the hemodynamic response function. The six movement parameters were included as covariates of no interest. Low frequency components were filtered out by applying a high-pass-filter with cut-off-frequency of 1/128 Hz. The error term was modeled as a first-order autoregressive process (AR coefficient = 0.2) plus white noise to account for serial autocorrelations within the data. The contrasts of emotional (happy and angry) versus neutral facial expressions obtained at first-level for each participant were used in a second-level random-effects analysis (one-sample t-test). Statistical significance was defined using a voxel-wise family-wise error (FWE) corrected height threshold of p < 0.05 and an extent threshold of k > 10 voxels. Assignment of brain structures to activated areas was based on the automated anatomical labelling (AAL) atlas^58^. To examine the impact of personality factors, correlation analyses between responses to emotional versus neutral stimuli in significantly activated brain regions (i.e. beta estimates across all voxels of activated clusters) and the five personality factors as obtained by the NEO-FFI were carried out (two-sided tests). Analogous analyses were carried out for the contrasts happy versus neutral and angry versus neutral to examine whether correlations found for the contrast emotional versus neutral are driven by one of the two conditions or occur similarly for both conditions. To allow for multiple comparisons a Bonferroni correction for the number of examined regions x five personality factors was employed.

Significant Pearson’s r correlation coefficients were further examined for effects of gender of the study participants and differential contributions of positive and negative emotions. To this end, we performed separate correlation analyses for female and male participants as well as emotional category (i.e. correlation between neuroticism and activation to positive versus neutral and negative versus neutral facial expressions). Resulting correlation coefficients were tested for significant differences between groups and conditions using t-tests. Finally, we tested whether correlation coefficients between brain activity and one of the five personality factor remain significant if the effects of the other four personality factors are regressed out. This was done using both simple regression analysis for each of the other four personality factors in isolation as well as multiple regression analysis to allow for potential conjoint effects of the four other personality factors.

Finally, we conducted exploratory whole-brain correlation analyses between brain activity to emotional versus neutral facial expressions and the five personality factors assessed by the NEO-FFI to enable inclusion of these data in future meta-analyses. The results of these correlation analyses are reported at a height threshold of p < 0.001 (uncorrected). Correction for multiple comparisons was carried out at cluster level (k > 90 voxels) corresponding to p < 0.05, corrected.

### Data availability

The datasets generated during and/or analysed during the current study are available from the corresponding author on reasonable request.
